# Real-World patterns of Korean medicine and combined Korean–Western medicine use in patients with chronic cough at Korean medicine institutions: a nationwide cohort study

**DOI:** 10.1186/s12906-026-05324-3

**Published:** 2026-03-06

**Authors:** Man Young Park, Beom-Joon Lee, Kwan-Il Kim, Yee Ran Lyu, Sung-Woo Kang, Boram Lee, Jun-Hwan Lee

**Affiliations:** 1 Digital Health Research Division, Korea Institute of Oriental Medicine, 1672 Yuseong-daero, Yuseong-gu, Daejeon, 34054 Republic of Korea; 2 Division of Allergy, Immune and Respiratory System, Department of Internal Medicine, College of Korean Medicine, Kyung Hee University, Kyung Hee University Medical Center, Seoul, 02447 Republic of Korea; 3 KM Science Research Division, Korea Institute of Oriental Medicine, 1672 Yuseong-daero, Yuseong-gu, Daejeon, 34054 Republic of Korea; 4 Department of Korean Medicine, College of Korean Medicine, Kyung Hee University, Seoul, 02447 Republic of Korea; 5 Korean Convergence Medical Science, KIOM School, University of Science & Technology (UST), Daejeon, 34054 Republic of Korea

**Keywords:** Chronic cough, Korean medicine, Integrative medicine, Cohort studies, Real-world evidence

## Abstract

**Background:**

Chronic cough impairs quality of life and increases healthcare utilization, yet no pharmacological treatment is formally approved. Korea’s dual healthcare system, which fully integrates Western medicine (WM) and Korean medicine (KM) under national insurance, provides a unique setting to examine real-world treatment patterns.

**Objective:**

To compare KM-only and integrative KM+WM care for chronic cough and describe the use of key KM modalities and multimodal treatment combinations.

**Methods:**

This retrospective cohort study analyzed nationwide claims data from the Health Insurance Review and Assessment Service (HIRA), 2011–2020. Chronic cough was defined as ≥56 days with ≥3 outpatient visits. Patients diagnosed with cough (R05) in KM institutions were included and classified into KM-only or KM+WM groups based on WM encounters within ±30 days of the index episode. KM modalities (acupuncture, moxibustion, cupping, herbal medicine) and WM prescriptions were assessed, with comorbidities evaluated using the Charlson Comorbidity Index (CCI).

**Results:**

Among 14,223 patients (mean age 58.6 years), 74.8% received KM-only care and 25.2% sought KM+WM care. Despite overall declines in KM utilization, the proportion of chronic cough patients among KM outpatients increased from 1.15% in 2011 to 2.76% in 2020. The KM+WM group had higher comorbidity burden (CCI ≥1: 43.7% vs. 32.8%) and more GERD (50.1% vs. 31.3%), allergic rhinitis (51.0% vs. 30.1%), and asthma (26.8% vs. 14.4%). Acupuncture was nearly universal (98.6%; mean 19.8 sessions), with cupping (59.3%) and hot–cold meridian therapy (42.9%) as common adjuncts. Herbal use was led by Samso-eum (35.0%) and So-cheong-ryong-tang (23.3%), whereas GERD-targeted formulas were rarely prescribed (Ojeok-san 3.3%, Saengmaek-san 2.7%).

**Conclusions:**

KM was the primary treatment option for chronic cough in Korea, while integrative KM+WM care was more common in patients with greater comorbidities. Acupuncture-centered multimodal approaches predominated, but the mismatch between GERD prevalence and GERD-targeted prescriptions highlights the need for standardized, evidence-based integrative guidelines. Findings reflect patients who initially sought KM care and should be interpreted within this context when informing policy and clinical practice.

**Supplementary Information:**

The online version contains supplementary material available at 10.1186/s12906-026-05324-3.

## Introduction

Chronic cough is more than a mere nuisance; it is associated with impaired quality of life, sleep disturbance, social withdrawal, and reduced productivity, and is increasingly recognized as an important global public health concern [1]. Common underlying causes include upper airway cough syndrome (often related to allergic rhinitis or chronic sinusitis), gastroesophageal reflux disease (GERD), and cough-variant asthma, which together account for the majority of chronic cough cases. In addition, drug-induced cough—most notably from angiotensin-converting enzyme (ACE) inhibitors—represents a well-recognized iatrogenic cause [2]. A recent systematic review reported that approximately 10% of adults worldwide experience chronic cough, with higher prevalence in Europe (12.7%) and North America (11.0%) compared to Asia (4.4%) [3, 4]. Nevertheless, substantial heterogeneity exists across Asian countries, and prevalence appears to be rising due to aging populations, environmental pollution, and lifestyle changes [5, 6]. In Korea, a national survey reported a chronic cough prevalence of 2.6% [7], and a recent comparative study between Korea and Taiwan found a 12-month prevalence of 4.34% in Korea [8], indicating that chronic cough constitutes a significant clinical burden in the Korean population. Thus, chronic cough contributes significantly to disease burden through diminished quality of life, increased medical expenditures, and heightened healthcare utilization [9, 10]. Among chronic diseases, chronic cough is particularly relevant to Korean Medicine (KM) practice, as traditional therapies such as acupuncture and herbal medicine have been widely used for respiratory conditions, and many patients with persistent or treatment-refractory symptoms turn to KM as a primary or complementary treatment option [11]. Despite this burden, no pharmacological treatment has been formally approved, and gaps remain between guideline-recommended management and real-world clinical practice [12–14]. The current treatment paradigm emphasizes identifying and addressing underlying etiologies (“cause-oriented treatment”). However, in up to 40–50% of patients, no clear cause can be determined, leading to empirical use of antihistamines, acid suppressants, neuromodulators, or complementary behavioral and physical therapies [15–17]. In this context, interest in traditional therapies such as acupuncture, herbal medicine, moxibustion, and cupping has increased, particularly among patients who do not experience sufficient improvement with Western medicine alone. Several studies have suggested that such complementary and integrative approaches may provide symptomatic relief and improve quality of life [11, 18, 19]. However, existing evidence is limited to small clinical trials or single-center studies, and there remains a critical lack of large-scale, nationwide data reflecting real-world treatment patterns. This gap is particularly significant because, without population-level evidence, it is impossible to determine how complementary and integrative approaches are actually utilized in routine clinical practice, which patient subgroups benefit most, or how treatment patterns compare across healthcare systems. Addressing this gap is essential for developing evidence-based clinical guidelines and informing healthcare policy. Korea provides a unique research setting as one of the few countries worldwide where both Western medicine (WM) and traditional Korean medicine (KM) are fully integrated into the National Health Insurance system [20, 21]. Patients can freely choose between, or combine, these two systems under a single coverage framework. This dual healthcare structure offers an exceptional opportunity to directly compare KM-only care with integrative KM–WM care in routine clinical practice [22]. Given these gaps in the literature and the unique advantages of Korea’s dual healthcare system, the present study analyzed nationwide claims data from 2011 to 2020 to investigate treatment patterns among patients with chronic cough who sought care at KM institutions. We focused on patients who initially presented to KM institutions rather than establishing a WM-only cohort, because our primary objective was to characterize treatment patterns within KM practice—an area that has been underexplored despite its clinical significance. This design allowed us to capture both patients who remained within KM care and those who additionally sought WM services, thereby illuminating the real-world utilization of integrative care from the KM perspective. Specifically, we aimed to: [1] compare patient characteristics between KM-only and KM + WM groups; [3] examine utilization of key KM modalities such as acupuncture, herbal medicine, moxibustion, and cupping alongside WM prescriptions; and [4] explore the patterns of multimodal treatment combinations in real-world practice. By doing so, this study provides evidence to inform clinical and policy decision-making regarding complementary and integrative approaches to chronic cough management.

## Methods

### Data source and study population

This retrospective cohort study was conducted using nationwide outpatient claims data from the Health Insurance Review and Assessment Service (HIRA) between January 1, 2011, and December 31, 2020. The study period was determined by the availability of customized HIRA research data, which requires a formal application process with an inherent lag of approximately 2–3 years between data submission and release; thus, 2020 was the most recent year available at the time of analysis. Notably, the inclusion of 2020 data allowed us to capture the initial impact of the COVID-19 pandemic on chronic cough patterns, although the full pandemic effects may extend beyond our observation period. Given the limitations of extracting data for all cough patients, we identified all patients who received a cough-related diagnosis code (R05) at Korean Medicine (KM) institutions. It should be noted that patients who received their initial cough diagnosis exclusively at WM institutions were not included, as the study was specifically designed to characterize treatment patterns among patients who sought KM care. For each patient, medical encounters were chronologically ordered by patient identifier and visit date, and repeated consultations for cough were grouped to define chronic cough episodes. No exclusions were made based on underlying diseases, in order to capture the comprehensive clinical spectrum of chronic cough and to reflect real-world treatment utilization. The study used customized research data from HIRA (project ID: M20230131001). As the dataset contained de-identified secondary data, the Institutional Review Board (IRB) granted an exemption from ethical review (IRB No. I-2301/001–002).

### Definition of chronic cough episodes

Cough episodes were reconstructed from claims records, with the start defined as the first visit date for a cough diagnosis. A sequence of visits was classified as a single episode if consecutive consultations were recorded without a gap exceeding 30 days. If no cough-related visits occurred for ≥ 30 days, the episode was considered terminated, and any subsequent visits were treated as a new episode. Chronic cough was defined as an episode lasting ≥ 56 days with at least three outpatient visits during that period. This operational definition is consistent with international clinical guidelines that define chronic cough as lasting ≥ 8 weeks [23, 24] and aligns with prior electronic medical record-based studies adopting a 56-day duration and ≥ 3 visits as a validated definition [25]. Although no external validation against clinical records was performed, the combination of duration and visit frequency thresholds has been shown to have high specificity for identifying true chronic cough cases in claims-based settings [25]. The requirement for at least three visits further reduces the likelihood of misclassification due to incidental or transient diagnoses.

### Classification of treatment groups

Healthcare provider type was classified using institutional codes in the claims database, distinguishing Korean Medicine (KM) institutions from Western Medicine (WM) institutions. For each patient, the observation window extended from 30 days before the index date (start of the representative episode) to 30 days after the end date. Patients with WM encounters within this window were categorized into the KM + WM (integrative care) group, while those without such encounters were assigned to the KM-only group. The ± 30-day window was chosen to capture patients who may have sought WM care shortly before or after their KM cough episode—for example, those who visited a WM provider for initial evaluation before seeking KM care, or those who subsequently consulted a WM provider for persistent symptoms. This window was considered clinically appropriate given typical healthcare-seeking intervals for chronic conditions. While longer windows (e.g., 60 or 90 days) could capture later WM encounters, the 30-day period was considered appropriate to identify patients who actively sought WM services in close temporal proximity to their KM care, rather than capturing incidental or unrelated WM visits that may occur during the prolonged course of chronic cough management. The potential impact of alternative window definitions on group classification is acknowledged as a limitation, and future sensitivity analyses using different time windows would further validate our classification approach. Because the cohort was constructed from patients who received cough diagnoses in KM institutions, the KM-only group represents the primary population, with the KM + WM group being a subset of the cohort.

### Treatment and medication data

Treatment data were linked across claims tables to identify both KM and WM interventions during chronic cough episodes. KM modalities included acupuncture, moxibustion, cupping, and herbal medicine, while WM prescriptions included antibiotics, antitussives/expectorants, antihistamines, and other medications. Only treatment and prescription records within the defined episode window were included for analysis.

### Comorbidity assessment

The Charlson Comorbidity Index (CCI) was calculated to evaluate the burden of comorbidities [26]. The CCI is a weighted index ranging from 1 to 6 points for 19 disease categories, widely used for mortality risk adjustment. We assessed each patient’s CCI based on diagnoses recorded during the two years prior to the index date.

### Statistical analysis

**Fig. 1:**
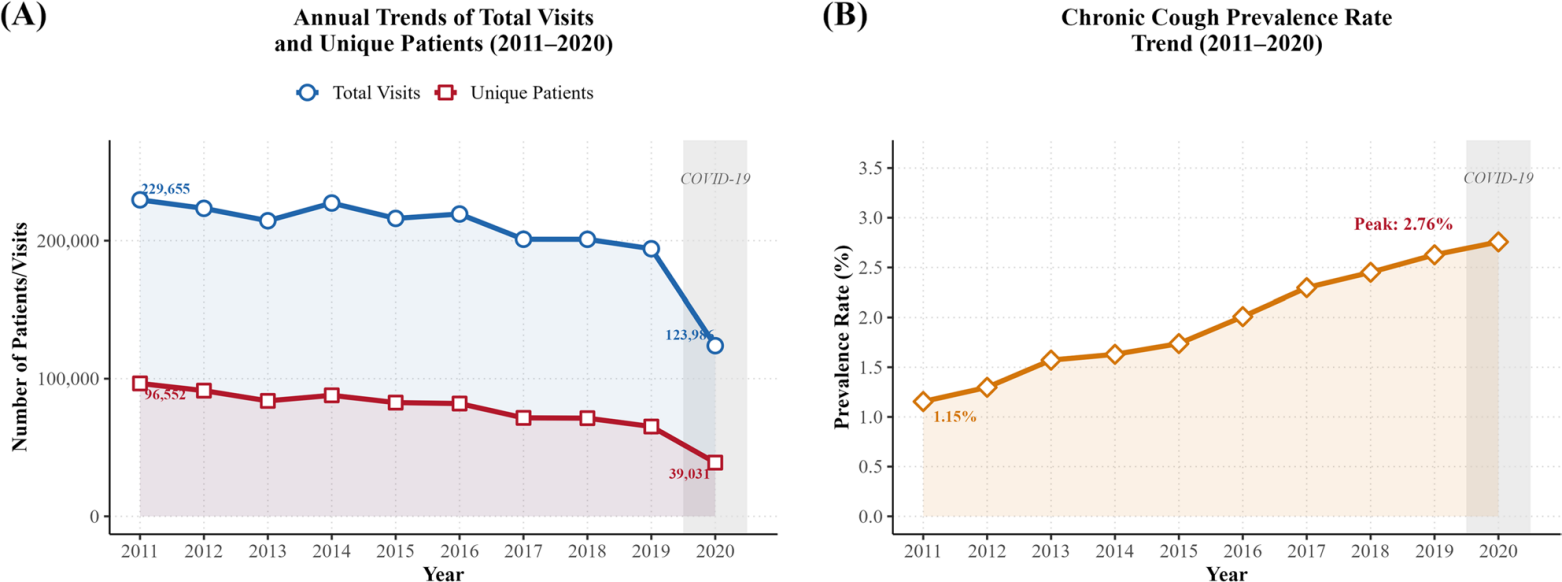
Annual trends in prevalence of chronic cough among KM outpatients, 2011–2020

Eligible patients were adults (≥ 18 years) with chronic cough episodes and at least one treatment or prescription record. The proportion of chronic cough patients among all KM outpatient visits was calculated annually to assess temporal trends. Demographic characteristics (age, sex) and clinical features (CCI, visit frequency, episode duration, treatment type) were compared between the KM-only and KM + WM groups. Descriptive statistics were presented as means with standard deviations (SD) for continuous variables and counts with percentages for categorical variables. Group comparisons were performed using chi-square tests or t-tests, as appropriate. Specifically, chi-square tests were used for categorical variables (sex, age groups, CCI categories, comorbidity prevalence), while independent-samples t-tests were used for continuous variables (mean age, number of visits, episode duration). All statistical tests were two-sided, and p-values < 0.05 were considered statistically significant. Regarding missing data, the HIRA claims database is a mandatory national reporting system, and all healthcare providers are required to submit claims for reimbursement. As such, the dataset has inherently minimal missing data for key variables, including diagnosis codes, procedure codes, and prescription records [27]. Variables used in this study (age, sex, diagnosis codes, treatment codes, institutional codes) had no missing values. The CCI was computed based on available diagnostic records; patients without any recorded comorbid diagnoses were assigned a CCI score of 0. All analyses were conducted using SAS (SAS Institute, Cary, NC, USA) and R (R Foundation for Statistical Computing, Vienna, Austria). ## Results ### Utilization of Korean Medicine and Trends in the Proportion of Chronic Cough Patients Between 2011 and 2020, overall utilization of Korean Medicine (KM) services showed a consistent decline. The total number of KM outpatient visits decreased from 229,655 in 2011 to 123,986 in 2020, representing a reduction of approximately 46%. Similarly, the number of unique patients declined by about 60%, from 96,552 in 2011 to 39,031 in 2020. These findings indicate a steady contraction in the overall scale of KM utilization over the past decade. In contrast, the proportion of chronic cough patients among all KM outpatients demonstrated a clear upward trend. The prevalence of chronic cough within the KM population was only 1.15% in 2011 but increased steadily each year, surpassing 2.0% in 2016 and reaching 2.76% by 2020. Notably, since 2017, the proportion has remained consistently above 2%, suggesting that the relative burden of chronic cough within KM practice has expanded, even as the overall patient population declined (Fig. 1).

A total of 14,223 patients met the inclusion criteria, of whom 10,638 (74.8%) were classified as the KM-only group and 3,585 (25.2%) as the KM + WM group (Table 1). The mean age of the overall cohort was 58.6 years, with more than half of the patients in both groups aged ≥ 50 years. The proportion of older patients was higher in the KM + WM group, in which 55.7% were aged ≥ 60 years, compared with 51.6% in the KM-only group (*p* < 0.001). Female patients predominated in both groups; however, the proportion of male patients was significantly higher in the KM-only group (30.2%) than in the KM + WM group (26.6%, *p* < 0.001). Patterns of healthcare utilization also differed between groups. The KM-only group had a higher mean number of outpatient visits (20.4 vs. 9.3) and a longer episode duration (101.9 vs. 84.4 days, *p* < 0.001) compared with the KM + WM group. With regard to comorbidity burden, patients with a Charlson Comorbidity Index (CCI) score ≥ 1 accounted for 43.7% of the KM + WM group, compared with 32.8% in the KM-only group. These findings indicate that patients receiving combined KM and WM care tended to have more comorbidities and a more complex clinical profile than those receiving KM care alone.

**Table 1:** Baseline characteristics of chronic cough patients by treatment group

Characteristics	KM only (*N* = 10,638)	KM + WM (*N* = 3,585)	Total (*N* = 14,223)	*p*-value
Age, mean ± SD	58.4 ± 15.4	59.3 ± 14.6	58.6 ± 15.2	0.001
Male sex, n (%)	3,209 (30.2)	952 (26.6)	4,161 (29.3)	< 0.001
Age group, n (%)				< 0.001
− 20–39 yrs	1,514 (14.2)	440 (12.2)	1,954 (13.7)	
− 40–59 yrs	3,635 (34.2)	1,148 (32.0)	4,783 (33.6)	
− 60–79 yrs	4,814 (45.3)	1,800 (50.2)	6,614 (46.5)	
- ≥80 yrs	675 (6.3)	197 (5.5)	872 (6.1)	
Visit count, mean ± SD	20.4 ± 21.8	9.3 ± 10.2	17.6 ± 20.1	< 0.001
Episode duration (days), mean ± SD	101.9 ± 78.1	84.4 ± 47.0	97.5 ± 72.0	< 0.001
Charlson comorbidity index, mean ± SD	0.5 ± 0.9	0.7 ± 1.0	0.5 ± 0.9	< 0.001
CCI category, n (%)				< 0.001
− 0	7,152 (67.2)	2,018 (56.3)	9,170 (64.5)	
− 1–2	3,082 (29.0)	1,364 (38.0)	4,446 (31.3)	
− 3–4	358 (3.4)	183 (5.1)	541 (3.8)	
- ≥5	46 (0.4)	20 (0.6)	66 (0.5)	

Continuous variables were compared using independent-samples t-tests; categorical variables were compared using chi-square tests

### Comorbidities and medication use in the three years prior to index date

Medication use during the three years prior to the index date showed markedly higher rates in the KM + WM group compared with the KM-only group (Table 2). Prescriptions for respiratory and allergy-related drugs were consistently more frequent in the KM + WM group, including corticosteroids (36.4% vs. 24.4%), antihistamines (31.4% vs. 19.0%), bronchodilators (34.5% vs. 21.5%), and expectorants/mucolytics (18.0% vs. 13.2%; all *p* < 0.001). Gastroprotective agents (41.3% vs. 29.3%) and antihypertensive medications (11.2% vs. 7.1%) were also more commonly prescribed in the KM + WM group, and use of antiallergic or immunomodulatory agents was nearly twice as high (7.1% vs. 3.6%). Comorbidity profiles, evaluated based on diagnostic records during the same three-year period, likewise indicated a greater burden among KM + WM patients. Allergic rhinitis (51.0% vs. 30.1%), asthma (26.8% vs. 14.4%), chronic bronchitis (20.7% vs. 11.0%), and chronic sinusitis (16.7% vs. 8.0%) were all significantly more prevalent in the KM + WM group (all *p* < 0.001). Gastroesophageal reflux disease (GERD) was diagnosed in more than half of KM + WM patients (50.1%) compared with 31.3% in the KM-only group (*p* < 0.001). In addition, major chronic conditions such as hypertension (27.3% vs. 21.1%) and diabetes mellitus (18.4% vs. 13.6%) were more common in the KM + WM group. By contrast, no significant between-group differences were observed for relatively rare conditions such as bronchiectasis, pulmonary fibrosis, or tuberculosis.

**Table 2:** Medication use and comorbidities within 3 years prior to the index date among chronic cough patients

Variables	KM only (*N* = 10,638)	KM + WM (*N* = 3,585)	Total (*N* = 14,223)	*p*-value
Medications				
Adrenal corticosteroids	2,600 (24.4)	1,305 (36.4)	3,905 (27.5)	< 0.001
Antihistamines	2,019 (19.0)	1,125 (31.4)	3,144 (22.1)	< 0.001
Bronchodilators	2,287 (21.5)	1,236 (34.5)	3,523 (24.8)	< 0.001
Expectorants & mucolytics	1,400 (13.2)	647 (18.0)	2,047 (14.4)	< 0.001
Anti-ulcer agents	3,113 (29.3)	1,481 (41.3)	4,594 (32.3)	< 0.001
Antihypertensives	752 (7.1)	401 (11.2)	1,153 (8.1)	< 0.001
Other allergy & immunology drugs	378 (3.6)	255 (7.1)	633 (4.5)	< 0.001
Comorbidities				
Allergic rhinitis	3,197 (30.1)	1,830 (51.0)	5,027 (35.3)	< 0.001
Asthma	1,536 (14.4)	960 (26.8)	2,496 (17.5)	< 0.001
Chronic bronchitis	1,166 (11.0)	743 (20.7)	1,909 (13.4)	< 0.001
Chronic sinusitis	847 (8.0)	598 (16.7)	1,445 (10.2)	< 0.001
GERD	3,327 (31.3)	1,797 (50.1)	5,124 (36.0)	< 0.001
Hypertension	2,241 (21.1)	978 (27.3)	3,219 (22.6)	< 0.001
Diabetes mellitus	1,443 (13.6)	660 (18.4)	2,103 (14.8)	< 0.001
Pneumonia	959 (9.0)	480 (13.4)	1,439 (10.1)	< 0.001
Heart failure	320 (3.0)	153 (4.3)	473 (3.3)	0.002

Values are expressed as mean ± standard deviation or number (%). Medication use and comorbidities were identified from claims records during the 3 years prior to the index date, including the index date

*KM only * Korean Medicine only,* KM+WM * Korean Medicine combined with Western Medicine, *GERD* gastroesophageal reflux disease

*p*-values were obtained using t-tests for continuous variables and chi-square tests for categorical variables

### Most frequently prescribed medications during the chronic cough period (Western Medicine)

During the chronic cough period, the most frequently prescribed Western medicine was expectorants/antitussives, which were used in 88.2% of patients, with an average of 6.3 prescriptions per patient (Table 3). Antihistamines were the second most common, prescribed to 66.9% of patients with a mean of 3.7 prescriptions. Gastroprotective agents (60.8%) and other antiallergic drugs (49.2%) were also prescribed in nearly half of the cohort. Other frequently used medications included opioid alkaloid antitussives (29.2%), bronchodilators and other respiratory agents (approximately 25%), otolaryngologic medications (28.2%), other gastrointestinal drugs (21.7%), and corticosteroids (25.4%). Psychotropic medications were prescribed to a smaller proportion of patients (8.4%), yet among those prescribed, the average number of prescriptions per patient was relatively high (4.6). Additional, less frequently used prescriptions are presented in Supplementary Table S1.

**Table 3:** Most frequently prescribed medications during chronic cough period (Western medicine)

Medication class	Total prescriptions	Unique patients (*N* = 3,325)	% of patients	Avg. per patient
Expectorants & mucolytics	18,373	2,933	88.20%	6.26
Antihistamines	8,297	2,225	66.90%	3.73
Anti-ulcer agents	6,648	2,020	60.80%	3.29
Other antiallergy drugs	5,416	1,635	49.20%	3.31
Opioid alkaloid antitussives	2,516	972	29.20%	2.59
Other respiratory drugs	2,097	849	25.50%	2.47
ENT preparations	1,980	938	28.20%	2.11
Other gastrointestinal drugs	1,979	721	21.70%	2.74
Adrenal corticosteroids	1,919	845	25.40%	2.27
Psychotropic agents	1,284	279	8.40%	4.6

Values are expressed as number or number (%). Data represent medications prescribed during the chronic cough period in Western medicine settings. The table shows the top 10 medication classes based on the proportion of patients who received each medication. Additional medications prescribed in fewer than 8% of patients are provided in Supplementary Table S1

### Utilization patterns of Korean medicine procedures during the chronic cough period

During the chronic cough period, acupuncture was by far the most frequently utilized Korean Medicine procedure. A total of 98.6% of patients received acupuncture, with an average of 19.8 sessions per patient, underscoring its role as the central therapeutic strategy (Table 4). Cupping therapy was performed in 59.3% of patients, with an average of 16.1 sessions per patient, making it the second most common modality. Hot and cold meridian therapy (a traditional Korean physical therapy modality involving thermal stimulation along meridian pathways) was administered to 42.9% of patients, with an average of 13.4 sessions, indicating its substantial use as an adjunctive treatment. In contrast, Chuna manual therapy (a traditional Korean manual therapy that combines spinal manipulation techniques with meridian theory, similar to chiropractic but based on traditional East Asian medicine principles) was rarely applied, being utilized in only 0.7% of patients. Overall, these findings demonstrate that acupuncture constituted the cornerstone of Korean Medicine management for chronic cough, while cupping and hot/cold meridian therapy were commonly used as complementary approaches, and Chuna was infrequently employed.

**Table 4:** Utilization patterns of Korean Medicine procedures during the chronic cough period

Treatment group	Total treatments	Unique patients (*N* = 11,151)	% of patients	Avg. per patient
Acupuncture	217,615	10,996	98.60%	19.8
Cupping	106,496	6,609	59.30%	16.1
Hot and cold meridian therapy	64,361	4,788	42.90%	13.4
Chuna	521	76	0.70%	6.9

This table summarizes the utilization patterns of Korean Medicine-based procedures among 11,151 patients with chronic cough. “Total procedures” indicates the overall number of sessions provided in each category. “Unique patients” refers to the number of patients who received the respective procedure at least once. “% of patients” represents the proportion of those patients out of the total cohort. “Avg. per patient” is calculated as the total number of procedures divided by the number of treated patients

### Most frequently prescribed herbal formulas during the chronic cough period

Analysis of herbal prescriptions during the chronic cough period revealed that a small number of formulas were used with notably high frequency. The most commonly prescribed formula was Samso-eum, which was given to 35.0% of patients, with an average of 8.3 prescriptions per patient (Table 5). This was followed by So-cheong-ryong-tang (23.3%), Ja-eum-ganghwa-tang (12.5%), and Haengso-tang (9.8%). Other frequently used prescriptions included Bojung-ikgi-tang, Ijin-tang, Yeongyo-paedok-san, and Gung-ha-tang. Interestingly, certain prescriptions such as Gung-ha-tang, Gamcho, and Jakyak were used in a relatively small proportion of patients, but those who received them had more than 15 prescriptions on average, indicating intensive use within specific patient subgroups. By contrast, many other herbal formulas were prescribed to less than 1% of patients and had minimal impact on overall frequency; these less common formulas are presented in Supplementary Table S2. Overall, the pattern of herbal prescriptions in chronic cough patients was characterized by predominant use of Samso-eum and So-cheong-ryong-tang, supplemented by a variety of traditional formulas tailored to individual clinical contexts.

**Table 5:** Top 15 herbal prescriptions used during the chronic cough period (Korean Medicine)

Prescription	Total prescription	Unique patients (*N* = 5,001)	% of patients	Avg. per patient
Samso-eum	14,576	1,750	35.00%	8.33
So-cheong-ryong-tang	9,493	1,167	23.30%	8.13
Ja-eum-ganghwa-tang	5,373	623	12.50%	8.62
Haengso-tang	3,709	490	9.80%	7.57
Bojung-ikgi-tang	2,409	263	5.30%	9.16
Ijin-tang	2,337	227	4.50%	10.3
Yeongyo-paedok-san	2,320	389	7.80%	5.96
Gung-ha-tang	2,191	145	2.90%	15.11
Gumi-ganghwal-tang	1,724	241	4.80%	7.15
Insam-paedok-san	1,433	243	4.90%	5.9
Gamcho (Glycyrrhizae Radix)	1,415	93	1.90%	15.22
Jakyak (Paeoniae Radix)	1,330	83	1.70%	16.02
Pyeongwi-san	1,323	191	3.80%	6.93
Ojeok-san	1,260	165	3.30%	7.64
Hyeonggae-yeongyo-tang	1,182	271	5.40%	4.36

This table presents the 15 most frequently prescribed herbal medicines during the chronic cough period among 5,001 patients. “Total prescriptions” indicates the total number of prescriptions for each formula. “Unique patients” refers to the number of patients who received the prescription at least once. “% of patients” represents the proportion of those patients out of the total cohort. “Avg. per patient” is calculated as the number of prescriptions divided by the number of treated patients. Additional prescriptions beyond the top 15 are provided in Supplementary Table S2

### Patterns of treatment combinations during the chronic cough period

During the chronic cough period, the most frequently observed treatment combination was acupuncture alone, accounting for 82,732 sessions among 4,536 patients (31.9% of the cohort), with an average of 18.2 sessions per patient (Table 6). The second most common pattern was acupuncture combined with cupping therapy, delivered in 42,503 sessions to 3,324 patients (23.4%), followed by acupuncture combined with cupping and hot and cold meridian therapy (28,723 sessions, 2,551 patients, 17.9%). Other frequently utilized multimodal regimens included acupuncture + cupping + herbal medicine (12.8% of patients) and acupuncture + hot and cold meridian therapy (10.5%). Notably, Western medicine alone was administered to 3,308 patients (23.3%), with a comparatively lower intensity (13,945 sessions, mean 4.2 sessions per patient). Pure herbal medicine prescriptions without procedures were observed in 1,607 patients (11.3%), with 10,568 sessions (mean 6.6 per patient). In addition, integrative regimens combining acupuncture, cupping, hot and cold meridian therapy, and herbal medicine were delivered to 1,362 patients (9.6%, 12,261 sessions). Overall, the findings indicate that while acupuncture formed the backbone of chronic cough management in Korean Medicine, it was frequently combined with cupping and other KM modalities, and in some cases integrated with herbal medicine or Western medicine.

**Table 6:** Treatment combinations including procedures, herbal medicine, and Western medicine during the chronic cough period5

Treatment combination	Total sessions	Unique patients (*N* = 14,223)	% of patients	Avg. sessions per patient
Acupuncture	82,732	4,536	31.90%	18.2
Acupuncture + Cupping	42,503	3,324	23.40%	12.8
Acupuncture + Cupping + Hot and cold meridian therapy	28,723	2,551	17.90%	11.3
Acupuncture + Cupping + Herbal medicine	19,365	1,824	12.80%	10.6
Acupuncture + Hot and cold meridian therapy	16,277	1,486	10.50%	11
Western medicine	13,945	3,308	23.30%	4.2
Acupuncture + Cupping + Hot and cold meridian therapy + Herbal medicine	12,261	1,362	9.60%	9
Herbal medicine	10,568	1,607	11.30%	6.6
Acupuncture + Herbal medicine	9,834	1,363	9.60%	7.2
Acupuncture + Hot and cold meridian therapy + Herbal medicine	5,304	783	5.50%	6.8

Values represent the most common treatment combinations. Less frequent combinations are presented in Supplementary Table S3

## Discussion

This large-scale retrospective cohort study analyzed 14,223 patients with chronic cough using nationwide claims data from the Korean National Health Insurance Service between 2011 and 2020. The key findings are as follows: First, the proportion of patients with chronic cough seeking Korean Medicine (KM) care increased steadily over the past decade, rising from 1.15% in 2011 to 2.76% in 2020, representing approximately a 2.4-fold increase. This trend highlights the expanding role of KM in the management of chronic cough within the national healthcare system.

Second, while the majority of patients (74.8%) received KM-only treatment, one-quarter (25.2%) sought combined care with Western Medicine (KM + WM). This pattern underscores the continued reliance on KM as a primary treatment option and reflects the unique dual healthcare system in Korea, which allows patients to freely choose between, or combine, KM and WM within the same insurance framework.

Third, patients in the KM + WM group exhibited a higher prevalence of comorbidities and greater medication use compared to those in the KM-only group. Notably, the prevalence of gastroesophageal reflux disease (GERD) was 50.1% in the KM + WM group versus 31.3% in the KM-only group, a statistically significant difference. These findings suggest that patients with greater disease complexity or comorbidity burden are more likely to seek integrative care.

Fourth, acupuncture was almost universally utilized, with 98.6% of patients receiving it at least once, confirming its role as the cornerstone of KM-based interventions for chronic cough. In contrast, adjunctive therapies such as moxibustion (42.9%) and cupping (59.3%) were used less frequently, indicating their role as complementary rather than primary interventions.

Finally, the analysis of treatment combinations (Table 6) revealed that greater diversity of therapies did not necessarily translate into more frequent visits. In some cases—such as the combination of acupuncture, cupping, and herbal medicine—the average number of treatments per patient was lower than that observed with single therapies. This finding suggests that treatment frequency in clinical practice is tailored according to patient symptomatology and therapeutic strategies, rather than being uniformly additive across modalities. Notably, the study population was predominantly female (approximately 70%) and older (mean age 58.6 years; >50% aged ≥ 60 years). These demographic characteristics are important potential confounders that should be considered when interpreting the results. The higher proportion of females may reflect known sex differences in cough sensitivity, as women have lower cough reflex thresholds and are more frequently affected by chronic cough than men [28]. Similarly, the predominance of older adults may reflect age-related increases in comorbidities (e.g., GERD, respiratory diseases) as well as generational preferences for KM among elderly Koreans. These factors could independently influence both treatment-seeking behavior and the observed patterns of KM utilization, and future studies should consider stratified analyses by sex and age to disentangle these effects.

### Implications of the increasing prevalence of chronic cough

The persistent increase in the prevalence of chronic cough observed over the 10-year study period (from 1.15% in 2011 to 2.76% in 2020, approximately a 2.4-fold rise) is noteworthy when compared with domestic and international epidemiological studies. A recent systematic review estimated the average prevalence of chronic cough in Asia at 4.4%, lower than in Europe (12.7%) or North America (11.0%) [3]. Although the 2020 prevalence in this study (2.76%) remained below the Asian average, it represented a clear upward trend relative to 2011. This suggests that the prevalence of chronic cough in Korea is gradually increasing. A previous study using the Korea National Health and Nutrition Examination Survey (KNHANES) reported a prevalence of 2.6% in 2010–2012 [7], which was higher than our estimates for the corresponding years (1.15–1.57%). While methodological differences and definitions must be considered, the overall trend of rising prevalence remains consistent. A recent comparative study between Korea and Taiwan also reported a 12-month prevalence of 4.34% in Korea [8], further supporting the long-term trajectory observed in our data. Several factors may contribute to this upward trend. First, chronic cough is strongly associated with aging, and Korea’s rapidly aging population is likely an important driver of prevalence growth. Second, worsening air pollution and other environmental factors may have exacerbated respiratory symptoms. Air pollution has previously been identified as a major risk factor for chronic cough in Korea [29]. Third, the rising prevalence of gastroesophageal reflux disease (GERD) may also play a role; in Korea, GERD prevalence increased from 7.1% in 2002 to 7.9% in 2007 [30], likely reflecting Westernized dietary and lifestyle changes. Fourth, improved access to healthcare and greater disease awareness may have led to higher diagnosis rates. Importantly, the COVID-19 pandemic in 2020 marked an exceptional turning point that warrants detailed consideration. Although overall healthcare utilization declined during the pandemic due to social distancing measures and avoidance of medical facilities, the prevalence of chronic cough peaked at 2.76% in our study. This paradoxical finding may be explained by several pandemic-related factors. First, the high prevalence of post-COVID cough—estimated at approximately 18% of recovered patients [31]—may have directly contributed to new chronic cough cases, particularly in the latter half of 2020. Second, changes in healthcare-seeking behavior during the pandemic may have altered the composition of patients presenting to KM institutions; patients with persistent respiratory symptoms who were unable to access conventional medical care or who feared COVID-19 exposure in hospital settings may have preferentially sought KM care. Third, pandemic-related lifestyle changes including prolonged mask wearing, social isolation, psychological stress, and reduced physical activity could have exacerbated cough-related symptoms or lowered symptom thresholds. However, as our data only extends through 2020, the full impact of COVID-19 on chronic cough epidemiology—including the emergence of long COVID and post-COVID cough syndromes—could not be fully captured, representing an important area for future investigation. The increasing prevalence of chronic cough among KM patients has important implications for public health policy. As the demand for KM-based respiratory care grows, healthcare systems should consider strengthening KM training programs with evidence-based protocols for chronic cough management. Furthermore, the integration of KM within national chronic respiratory disease management strategies could help address the unmet clinical needs of patients who do not respond adequately to conventional therapies. These findings also support the development of interdisciplinary clinical pathways that facilitate coordinated KM–WM care for chronic cough patients, particularly those with complex comorbidity profiles.

### High proportion of korean medicine-only utilization and its implications

This cohort was constructed from patients diagnosed with cough (R05) in Korean Medicine (KM) clinics, and 74.8% of the study population received KM-only treatment. This finding indicates that a substantial proportion of chronic cough patients who initially sought KM care continued to rely exclusively on this system. Thus, the results should not be generalized to all chronic cough patients, but rather interpreted as reflecting the characteristics of those who selected KM care. Given the relatively older mean age of the study population (~ 60 years), the higher KM utilization may partly reflect the treatment preferences of older adults, who are known to favor KM. Furthermore, KM’s holistic approach and perceived lower risk of adverse effects likely make it an attractive option for patients with complex, multifactorial symptoms. However, as this study relied on claims data, non-reimbursed KM treatments and over-the-counter medications were not captured. Therefore, some patients categorized as KM-only users may in fact have received WM in parallel, and this should be taken into account when interpreting the findings. Future studies incorporating survey data or qualitative approaches will be valuable in more accurately characterizing real-world treatment behaviors.

### Differences in patient characteristics between treatment groups

Clear differences were observed between the KM-only and KM + WM groups, reflecting distinct levels of clinical complexity. Patients in the KM + WM group had higher prevalences of major comorbidities (GERD 50.1% vs. 31.3%, allergic rhinitis 51.0% vs. 30.1%, asthma 26.8% vs. 14.4%), greater prescription rates for key medications, and higher CCI scores. Conversely, KM-only patients had longer episode durations (101.9 vs. 84.4 days) and more frequent visits (20.4 vs. 9.3), suggesting sustained KM utilization in less complex cases. This pattern may indicate that patients with milder or less complicated disease profiles tend to rely on KM for long-term symptom management, where repeated visits are intended to maintain or gradually improve symptoms rather than to address acute exacerbations. Overall, these findings suggest that patients with greater clinical complexity were more likely to seek integrative care, whereas those with milder presentations tended to rely on KM alone. Notably, the markedly higher prevalence of GERD in the KM + WM group (50.1% vs. 31.3%) highlights the importance of GERD as a common comorbidity in chronic cough. Because GERD-related cough often requires pharmacological therapy and remains challenging to manage effectively, this observation underscores a potential treatment gap in which KM interventions alone may be insufficient, thereby driving patients toward integrative KM–WM care.

### Patterns and clinical implications of korean medicine and integrative interventions

Acupuncture was administered to nearly all patients (98.6%), confirming its role as the cornerstone of KM-based treatment for chronic cough. The average of 19.8 sessions per patient, spanning approximately 3–4 months based on typical treatment schedules, suggests that chronic cough requires sustained neuromodulatory intervention rather than short-term symptomatic relief. This finding aligns with systematic reviews and meta-analyses reporting that acupuncture-related therapies significantly improve cough severity and quality of life through cumulative therapeutic effects [18]. Mechanistically, acupuncture is thought to modulate the cough reflex through peripheral and central nervous system pathways, including downregulation of transient receptor potential (TRP) channels, reduction of airway neurogenic inflammation, and modulation of vagal afferent signaling [32]. Cupping therapy (59.3%) may contribute to symptom relief through local vasodilation, promotion of lymphatic drainage, and reduction of muscle tension in the thoracic and upper back regions, thereby potentially improving respiratory mechanics [33]. Herbal medicine formulations such as Samso-eum and So-cheong-ryong-tang contain multiple bioactive compounds with documented anti-inflammatory, antitussive, and immunomodulatory properties. For example, So-cheong-ryong-tang has been shown in clinical studies to reduce cough frequency and severity in patients with allergic rhinitis and upper airway cough syndrome [34]. Cupping therapy (59.3%) and hot–cold meridian therapy (42.9%) were frequently applied as adjunctive modalities, with average session counts of 16.1 and 13.4 respectively, reflecting the multimodal approach characteristic of KM practice. In contrast, Chuna manual therapy was rarely utilized (0.7%), indicating its selective application in specific clinical contexts rather than routine use for chronic cough management. Herbal medicine prescriptions were dominated by Samso-eum (35.0%) and So-cheong-ryong-tang (23.3%), both traditionally indicated for cough-related conditions. The frequent use of So-cheong-ryong-tang is particularly noteworthy given the high prevalence of allergic rhinitis in our cohort (51.0% in KM + WM group, 30.1% in KM-only group), as this formula has documented efficacy in allergic respiratory conditions [34]. Other commonly prescribed formulas included Ja-eum-ganghwa-tang (12.5%) and Haengso-tang (9.8%), reflecting the heterogeneous clinical presentations requiring individualized treatment approaches. A notable finding was the infrequent prescription of formulas specifically indicated for GERD-related cough, such as Ojeok-san (3.3%) and Saengmaek-san (2.7%), despite GERD being present in 50.1% of the KM + WM group and 31.3% of the KM-only group. This discrepancy suggests either underutilization of targeted therapies or the absence of well-established, evidence-based KM protocols for GERD-associated chronic cough, highlighting an area for future clinical guideline development. Analysis of treatment combinations revealed that acupuncture alone accounted for the largest proportion of patients (31.9%), followed by acupuncture with cupping (23.4%) and acupuncture combined with cupping plus hot–cold meridian therapy (17.9%). However, these patterns should be interpreted with caution, as the claims database captures only reimbursed treatments. Non-reimbursed herbal prescriptions, commonly used in routine KM practice, were not included, potentially leading to misclassification of some patients as receiving “acupuncture alone” when they may have received comprehensive integrative care. This limitation underscores a broader methodological challenge in KM research and highlights the need for data collection systems that can capture the full spectrum of integrative therapeutic approaches.

### Strengths and limitations

This study has several notable strengths. First, it is a large-scale, nationwide cohort study utilizing the comprehensive HIRA claims database within a single-payer system, ensuring representativeness and minimizing selection bias. Second, unlike most previous studies focused on WM institutions, this study uniquely constructed its cohort from KM institutions, illuminating underexplored clinical behaviors within Korea’s dual healthcare system. Third, the decade-long observation period captured temporal changes in chronic cough prevalence, including the initial impact of the COVID-19 pandemic. Fourth, systematic comparisons of patient characteristics, comorbidities, and treatment patterns between KM-only and KM + WM groups enhance the clinical relevance of the findings. However, this study also has limitations. First, owing to the nature of claims data, non-reimbursed KM treatments and over-the-counter medications were not captured, potentially leading to misclassification of treatment patterns and overestimation of KM-only utilization. Second, because the cohort was constructed from patients diagnosed with cough (R05) at KM institutions, our findings may not be generalizable to all chronic cough patients in Korea, and KM utilization may be overrepresented relative to the general chronic cough population. Third, COVID-19 was not included as a separate comorbidity variable, as it emerged only in the final year of the study period; a follow-up study examining the relationship between COVID-19 and KM utilization patterns is being planned. Fourth, as a retrospective analysis, patient-centered outcomes such as treatment effectiveness, symptom severity, quality of life, and patients’ motivations for treatment choice could not be assessed. Finally, alternative time windows for classifying treatment groups and sensitivity analyses were not performed, which should be addressed in future studies. Future research should prioritize RCTs evaluating specific KM modalities for chronic cough, studies incorporating validated patient-centered outcome measures, and the development of standardized KM treatment protocols for common comorbidities such as GERD and allergic rhinitis.

## Conclusion

In this nationwide cohort of over 14,000 patients with chronic cough, nearly three-quarters received Korean Medicine (KM) alone, underscoring KM’s role as a primary treatment option. Patients who sought combined KM and Western Medicine (WM) care had greater comorbidity burdens and more complex medication profiles, reflecting the clinical need for integrative approaches. Acupuncture was almost universally used, while cupping, hot–cold meridian therapy, and herbal prescriptions were frequent adjuncts. These findings highlight the real-world significance of KM in chronic cough management and suggest future policies and guidelines should better integrate KM within comprehensive care pathways, especially for patients with persistent or unexplained chronic cough. However, as this study was limited to patients who received a cough diagnosis at KM institutions, the findings may not be generalizable to all chronic cough patients, and future studies should include patients from WM institutions to provide a more comprehensive picture.

## Supplementary Information

Supplementary Material 1.

## Data Availability

The data underlying this article are not publicly available due to restrictions from the Health Insurance Review and Assessment Service (HIRA). Access to these data requires approval from HIRA (https://opendata.hira.or.kr), and the authors are not permitted to share the data directly.
