# Supplementary material for: Real-World patterns of Korean medicine and combined Korean–Western medicine use in patients with chronic cough at Korean medicine institutions: a nationwide cohort study
Source: BMC Complement Med Ther. 2026 Mar 6;26:138. doi: 10.1186/s12906-026-05324-3 (PMC13078034; doi:10.1186/s12906-026-05324-3)
Supplement: Supplementary file 1 — Supplementary Material 1. [file 12906_2026_5324_MOESM1_ESM.docx]

Supplementary Table S1. Additional medications prescribed during chronic cough period (Western medicine)

| Medication class | Total prescriptions | Unique patients  (N=3,325) | % of patients | Avg. per patient |
| --- | --- | --- | --- | --- |
| Antibiotics (Gram + / Gram –) | 1,246 | 717 | 21.60% | 1.74 |
| Antipyretic, analgesic, anti-inflammatory | 896 | 441 | 13.30% | 2.03 |
| Other chemotherapeutics | 625 | 354 | 10.70% | 1.77 |
| Antibiotics (Gram +, rickettsia, virus) | 420 | 258 | 7.80% | 1.63 |
| Other respiratory drugs (unspecified) | 404 | 166 | 5.00% | 2.43 |
| Enzyme preparations | 377 | 166 | 5.00% | 2.27 |
| X-ray contrast media | 372 | 349 | 10.50% | 1.07 |
| Antacids | 325 | 132 | 4.00% | 2.46 |
| Other antibiotics (combinations) | 261 | 130 | 3.90% | 2.01 |
| Other hematology/fluid drugs | 183 | 140 | 4.20% | 1.31 |
| Antihypertensives | 157 | 60 | 1.80% | 2.62 |
| Local anesthetics | 148 | 98 | 3.00% | 1.51 |
| Plasma expanders | 148 | 64 | 1.90% | 2.31 |
| Anti-arteriosclerotic agents | 135 | 48 | 1.40% | 2.81 |
| Hypnotics/sedatives | 117 | 97 | 2.90% | 1.21 |
| Hepatic agents | 114 | 24 | 0.70% | 4.75 |
| Dental/oral preparations | 110 | 70 | 2.10% | 1.57 |
| Spasmolytics | 109 | 83 | 2.50% | 1.31 |
| Antituberculosis drugs | 99 | 12 | 0.40% | 8.25 |
| Diuretics | 88 | 27 | 0.80% | 3.26 |
| Antidiabetics | 82 | 18 | 0.50% | 4.56 |
| Other metabolic drugs | 54 | 24 | 0.70% | 2.25 |
| Minerals | 48 | 13 | 0.40% | 3.69 |
| Muscle relaxants | 42 | 14 | 0.40% | 3 |
| Vitamins (B, A, D, C) | 59 | 18 | 0.50% | 2.28 |
| Hemostatic agents | 33 | 21 | 0.60% | 1.57 |
| Others (rare prescriptions*) | <30 each | <1% | – | – |

Supplementary Table S2. Additional Herbal prescriptions used during the chronic cough period (Korean Medicine)

| Prescription | Total procedures | Unique patients  (N=5,001) | % of patients | Avg. per patient |
| --- | --- | --- | --- | --- |
| Sosihotang | 1,085 | 190 | 3.80% | 5.71 |
| Saengmaeksan | 988 | 133 | 2.70% | 7.43 |
| Galgeun-tang | 936 | 170 | 3.40% | 5.51 |
| Hyangsa-pyeongwi-san | 762 | 161 | 3.20% | 4.73 |
| Banha-sasim-tang | 665 | 119 | 2.40% | 5.59 |
| Banha-hobak-tang | 584 | 118 | 2.40% | 4.95 |
| Bulhwan-geumjeonggi-san | 512 | 124 | 2.50% | 4.13 |
| Sigyeong-banha-tang | 408 | 76 | 1.50% | 5.37 |
| Baekchul-tang | 305 | 27 | 0.50% | 11.3 |
| Palmul-tang | 297 | 32 | 0.60% | 9.28 |
| Daeju (Zizyphi Fructus) | 275 | 19 | 0.40% | 14.47 |
| Galgeun-haegi-tang | 267 | 52 | 1.00% | 5.13 |
| Dokhwal (Angelicae Pubescentis Radix) | 233 | 10 | 0.20% | 23.3 |
| Hwangryeon-haedok-tang | 212 | 54 | 1.10% | 3.93 |
| Saenggang (Zingiberis Rhizoma) | 192 | 10 | 0.20% | 19.2 |
| Gami-soyo-san | 185 | 59 | 1.20% | 3.14 |
| Gyeji (Cinnamomi Ramulus) | 181 | 8 | 0.20% | 22.62 |
| Naeso-san | 170 | 33 | 0.70% | 5.15 |
| Banha-baekchul-cheonma-tang | 152 | 56 | 1.10% | 2.71 |
| Ijung-tang | 142 | 25 | 0.50% | 5.68 |
| Siho-gyeji-tang | 131 | 35 | 0.70% | 3.74 |
| Ganghwal (Osterici Radix) | 124 | 9 | 0.20% | 13.78 |
| Bangpung (Saposhnikoviae Radix) | 122 | 7 | 0.10% | 17.43 |
| Danggui (Angelicae Gigantis Radix) | 120 | 11 | 0.20% | 10.91 |
| Maekmundong (Ophiopogonis Radix) | 110 | 12 | 0.20% | 9.17 |
| Insam (Ginseng Radix) | 110 | 11 | 0.20% | 10 |
| Banha (Pinelliae Tuber) | 103 | 10 | 0.20% | 10.3 |
| Cheongsang-gyeontong-tang | 92 | 19 | 0.40% | 4.84 |
| Sukjihwang (Rehmanniae Radix Preparata) | 84 | 5 | 0.10% | 16.8 |
| Sansa (Crataegi Fructus) | 79 | 3 | 0.10% | 26.33 |
| Doin-seunggi-tang | 58 | 15 | 0.30% | 3.87 |
| Daecheongryong-tang | 55 | 22 | 0.40% | 2.5 |
| Jaso-yeop (Perillae Folium) | 54 | 3 | 0.10% | 18 |
| Samchul-geonbi-tang | 53 | 19 | 0.40% | 2.79 |
| Haengin (Armeniacae Semen) | 48 | 5 | 0.10% | 9.6 |
| Changchul (Atractylodis Rhizoma) | 47 | 4 | 0.10% | 11.75 |
| Orim-san | 45 | 6 | 0.10% | 7.5 |
| Mahwang (Ephedrae Herba) | 42 | 3 | 0.10% | 14 |
| Seokgo (Gypsum Fibrosum) | 37 | 2 | 0.00% | 18.5 |
| Jeonho (Peucedani Radix) | 37 | 3 | 0.10% | 12.33 |
| Gilgyeong (Platycodi Radix) | 36 | 5 | 0.10% | 7.2 |
| Yeongyo (Forsythiae Fructus) | 36 | 2 | 0.00% | 18 |
| Hyeonggae (Schizonepetae Spica) | 36 | 2 | 0.00% | 18 |
| Daesiho-tang | 35 | 6 | 0.10% | 5.83 |
| Jowi-seunggi-tang | 33 | 10 | 0.20% | 3.3 |
| Jisil (Aurantii Fructus Immaturus) | 32 | 1 | 0.00% | 32 |
| Daehwa-jung-eum | 31 | 9 | 0.20% | 3.44 |
| Bakha (Menthae Herba) | 29 | 2 | 0.00% | 14.5 |
| Hoechun-yang-gyeok-san | 29 | 7 | 0.10% | 4.14 |
| Gwallu-in (Trichosanthis Semen) | 28 | 1 | 0.00% | 28 |
| Saengjihwang (Rehmanniae Radix) | 28 | 1 | 0.00% | 28 |
| Siho (Bupleuri Radix) | 28 | 1 | 0.00% | 28 |
| Singok (Massa Medicata Fermentata) | 28 | 2 | 0.00% | 14 |
| Maek-a (Hordei Fructus Germinatus) | 27 | 1 | 0.00% | 27 |
| Hwanggi (Astragali Radix) | 25 | 2 | 0.00% | 12.5 |
| Baekchul (Atractylodis Macrocephalae Rhizoma) | 23 | 3 | 0.10% | 7.67 |
| Cheongung (Cnidii Rhizoma) | 21 | 2 | 0.00% | 10.5 |
| Jinpi (Citri Reticulatae Pericarpium) | 20 | 5 | 0.10% | 4 |
| Cheongseo-ikgi-tang | 18 | 5 | 0.10% | 3.6 |
| Ikwi-seungyang-tang | 16 | 5 | 0.10% | 3.2 |
| Bokryeong (Poria) | 15 | 3 | 0.10% | 5 |
| Sesin (Asari Radix) | 15 | 1 | 0.00% | 15 |
| Jimo (Anemarrhenae Rhizoma) | 15 | 1 | 0.00% | 15 |
| Cheonma (Gastrodiae Rhizoma) | 15 | 1 | 0.00% | 15 |
| Hwangbaek (Phellodendri Cortex) | 15 | 1 | 0.00% | 15 |
| Injinho-tang | 14 | 4 | 0.10% | 3.5 |
| Gwakhyang (Agastachis Herba) | 10 | 1 | 0.00% | 10 |
| Bokryeong-bosim-tang | 10 | 6 | 0.10% | 1.67 |
| Geumunhwa (Lonicerae Flos) | 8 | 2 | 0.00% | 4 |
| Omija (Schisandrae Fructus) | 8 | 3 | 0.10% | 2.67 |
| Gungso-san | 7 | 2 | 0.00% | 3.5 |
| Samhwang-sasim-tang | 7 | 3 | 0.10% | 2.33 |
| Cheonmundong (Asparagi Tuber) | 7 | 2 | 0.00% | 3.5 |
| Daehwang-mokdanpi-tang | 4 | 2 | 0.00% | 2 |
| Siho-cheonggan-tang | 4 | 3 | 0.10% | 1.33 |
| Galgeun (Puerariae Radix) | 2 | 2 | 0.00% | 1 |
| Boheo-tang | 2 | 2 | 0.00% | 1 |
| Siho-sogan-tang | 2 | 2 | 0.00% | 1 |
| Hyangbuja | 2 | 2 | 0.00% | 1 |
| Gyeonggang (Zingiberis Rhizoma Crudus) | 1 | 1 | 0.00% | 1 |
| Doin (Persicae Semen) | 1 | 1 | 0.00% | 1 |
| Ji-gak (Aurantii Fructus) | 1 | 1 | 0.00% | 1 |
| Chija (Gardeniae Fructus) | 1 | 1 | 0.00% | 1 |
| Hwanggeum (Scutellariae Radix) | 1 | 1 | 0.00% | 1 |
| Hubak (Magnoliae Cortex) | 1 | 1 | 0.00% | 1 |
| Ai-world Yukgye | 1 | 1 | 0.00% | 1 |

Supplementary Table S3. Additional treatment combinations during the chronic cough period

| Treatment combination | Total sessions | Unique patients (N=14,223) | % of patients | Avg. sessions per patient |
| --- | --- | --- | --- | --- |
| Cupping | 1,783 | 185 | 1.30% | 9.6 |
| Cupping + Hot and cold meridian therapy | 962 | 104 | 0.70% | 9.3 |
| Acupuncture + Chuna + Cupping + Hot and cold meridian therapy | 379 | 51 | 0.40% | 7.4 |
| Cupping + Herbal medicine | 209 | 68 | 0.50% | 3.1 |
| Cupping + Hot and cold meridian therapy + Herbal medicine | 151 | 36 | 0.30% | 4.2 |
| Hot and cold meridian therapy | 109 | 37 | 0.30% | 3 |
| Hot and cold meridian therapy + Herbal medicine | 99 | 33 | 0.20% | 3 |
| Acupuncture + Chuna + Cupping | 52 | 9 | 0.10% | 5.8 |
| Acupuncture + Chuna + Cupping + Hot and cold meridian therapy + Herbal medicine | 42 | 13 | 0.10% | 3.2 |
| Acupuncture + Western Medicine | 36 | 32 | 0.20% | 1.1 |
| Acupuncture + Cupping + Western Medicine | 25 | 24 | 0.20% | 1 |
| Acupuncture + Cupping + Hot and cold meridian therapy + Western Medicine | 20 | 17 | 0.10% | 1.2 |
| Acupuncture + Chuna | 16 | 4 | 0.00% | 4 |
| Acupuncture + Chuna + Hot and cold meridian therapy + Herbal medicine | 10 | 3 | 0.00% | 3.3 |
| Acupuncture + Hot and cold meridian therapy + Western Medicine | 8 | 8 | 0.10% | 1 |
| Acupuncture + Chuna + Cupping + Herbal medicine | 7 | 5 | 0.00% | 1.4 |
| Acupuncture + Cupping + Herbal medicine + Western Medicine | 6 | 5 | 0.00% | 1.2 |
| Acupuncture + Chuna + Hot and cold meridian therapy | 4 | 2 | 0.00% | 2 |
| Acupuncture + Chuna + Herbal medicine | 3 | 2 | 0.00% | 1.5 |
| Acupuncture + Cupping + Hot and cold meridian therapy + Herbal medicine + Western Medicine | 3 | 3 | 0.00% | 1 |
| Acupuncture + Hot and cold meridian therapy + Herbal medicine + Western Medicine | 3 | 3 | 0.00% | 1 |
| Chuna + Hot and cold meridian therapy | 3 | 2 | 0.00% | 1.5 |
| Chuna + Cupping + Hot and cold meridian therapy | 2 | 2 | 0.00% | 1 |
| Herbal medicine + Western Medicine | 2 | 2 | 0.00% | 1 |
| Acupuncture + Chuna + Cupping + Hot and cold meridian therapy + Western Medicine | 1 | 1 | 0.00% | 1 |
| Acupuncture + Herbal medicine + Western Medicine | 1 | 1 | 0.00% | 1 |
| Chuna | 1 | 1 | 0.00% | 1 |
| Chuna + Cupping + Herbal medicine | 1 | 1 | 0.00% | 1 |
| Cupping + Western Medicine | 1 | 1 | 0.00% | 1 |
